# The native European *Aedes geniculatus* mosquito species can transmit chikungunya virus

**DOI:** 10.1080/22221751.2019.1634489

**Published:** 2019-07-01

**Authors:** Jorian Prudhomme, Albin Fontaine, Guillaume Lacour, Jean-Charles Gantier, Laure Diancourt, Enkelejda Velo, Silva Bino, Paul Reiter, Aurélien Mercier

**Affiliations:** a UMR MIVEGEC, IRD 224, CNRS 5290, Université de MontpellierMontpellier, France; b Unité de Parasitologie et Entomologie, Institut de Recherche Biomédicale des Armées (IRBA), Marseille, France; c IRD, AP-HM, SSA, UMR Vecteurs – Infections Tropicales et Méditerranéennes (VITROME), IHU - Méditerranée Infection, Aix Marseille Université, Marseille, France; d Unité Contrôle et Adaptation des Vecteurs, Institut Pasteur de la Guyane, Cayenne, France; e Laboratoire des Identiﬁcations Fongiques et Entomo-parasitologiques, Mennecy, France; f Genotyping of Pathogens and Public Health, Institut Pasteur, Paris, France; g Control of Infectious Diseases Department, Institute of Public Health, Tirana, Albania; h Unité Insectes et Maladies Infectieuses, Institut Pasteur, Paris, France; i INSERM, U1094, Neuroépidémiologie Tropicale, Limoges, France

**Keywords:** *Aedes geniculatus*, *Aedes albopictus*, chikungunya, Europe, vector competence, autochtonous species, arbovirus, globalization

## Abstract

Europe is the world’s leading tourism destination and is receiving every year travellers from areas with active arbovirus transmission. There is thus a threat of mosquito-borne virus emergence in Europe due to the presence of the invasive mosquito vector *Aedes albopictus*. Little attention has been paid about the possible role of indigenous mosquito species as vectors of emerging arboviruses. Here, we assessed the vector competence dynamic of *Aedes geniculatus*, a European anthropophilic mosquito species, for chikungunya virus (CHIKV) in comparison with an European population of *Ae. albopictus*. We revealed that *Ae. geniculatus* is highly susceptible to CHIKV infection and could transmit the virus. By specifically exploring the vector competence dynamic in both mosquito species, we revealed that the cumulative distribution of CHIKV incubation period in *Ae. geniculatus* was delayed by several days as compared to *Ae. albopictus*. Our results strengthen the importance of considering indigenous species as potential vectors for emerging arboviruses. They also revealed the importance of considering variation in arbovirus dissemination or transmission dynamics in mosquitoes when performing vector competence assays. We will discuss the implications of our results on a CHIKV outbreak dynamic in a theoretical framework.

## Introduction

Chikungunya virus (CHIKV) is a mosquito-borne virus that has emerged from its sylvatic habitat in Africa and is now transmitted in many urban regions world-wide wherever competent vectors, primarily *Aedes aegypti* and *Aedes albopictus,* are present. Three distinct lineages of CHIKV are sporadically causing outbreaks in human population [1,2].

First detected in Albania and later in Italy, the *Ae. albopictus* species [3] is now established throughout much of the Mediterranean basin and northward as far as Paris [4]. The ever-increasing air travel between Europe and regions with active arbovirus transmission, in combination with travel times that are largely inferior to the incubation period, has increased the risk of arboviruses introduction into Europe, as illustrated by transmission events of CHIKV in several European countries [5–8]. For example, an outbreak of CHIKV in Italy in 2017 involved more than 420 confirmed cases [5].

*Aedes albopictus* has been implicated as the vector for all European CHIKV outbreaks but an indigenous species, *Aedes geniculatus* (Olivier, 1791) is frequently found in ovitraps used for surveillance of *Ae. albopictus* in outbreak areas [9]. Both are tree-hole species that breed in natural containers in woodland as well as in man-made containers in the peri-urban and peri-domestic environment [10,11]. Their eggs are resistant to desiccation and can overwinter in temperate areas. Both species are day-active, exophilic and feed aggressively on humans and other mammals. The implication of European mosquitoes in the transmission of emerging arboviruses has been poorly investigated despite evidence of transmission by other *Aedes* species outside Europe [2]. We compared the competence of the native European mosquito species *Ae. geniculatus* to transmit CHIKV in comparison with the invasive and reference vector species for CHIKV: *Ae. albopictus*. Importantly, the consideration of the dynamic nature of vector competence revealed that differences of vector competence between mosquito species were due to a time shift in the distribution of extrinsic incubation periods rather than differences in maximum proportion of infectious mosquitoes. The importance of considering indigenous species as potential vectors for arbovirus and the implication, in a theoretical framework, of our results on a CHIKV outbreak dynamic would be discussed.

## Materials and Methods

### Mosquito collection and identification

Mosquitoes from both *Ae. albopictus* and *Ae. geniculatus* species used in this study originated in Tirana, the capital of Albania. Eggs were collected by ovitrap in an urban park (41° 18’36” N; 19° 49’18” E) from July to August 2012. Eggs from both species were hatched at the Institut Pasteur in Paris and reared under standard conditions. Adults from the F0 generation were identified morphologically [12] and 500 individuals of each species were placed per cage at 26°C ± 1°C with 60–70% relative humidity and a light: dark cycle of 16 h: 8 h. These temperature and humidity rate values were recorded throughout the rearing process with an electronic sensor. Adults were given 10% sucrose solution and females were allowed to engorge with rabbit blood on a membrane feeding apparatus (Hemotek, Discovery Workshops, Lancashire, United Kingdom) to obtain F1 eggs. Batches of eggs were hatched simultaneously to obtain females of the same physiological age for experimental infections. Larvae were reared to the adult stage under the same conditions. All experiments were realized with the F1 generation.

### Virus

The isolate CHIKV 0621 was used in this study. This viral strain was isolated from a traveller from India and possesses an amino acid change (A226 V) in the envelope glycoprotein E1 [13–15] [GenBank accession number DQ443544]. The virus had been passaged three times in C6/36 cells prior to use in the experiments. Virus titration was performed by focus-forming assay (FFA) as previously described [16]. The titre of the frozen virus stock was estimated as 10^9^ focus-forming units per mL (FFU/mL). All infectious experiments were conducted in a BSL-3 insectary (Institut Pasteur, Paris).

### Mosquito infections

Females 9–10 days old were deprived of sucrose solution 24 h before experimental infection. The infectious blood-meal consisted of 1 mL of viral suspension, 2 mL of washed rabbit erythrocytes supplemented with adenosine triphosphate (10 mM) as a phagostimulant. Blood feeding was by an artificial feeding apparatus (Hemotek) covered with pig intestine. The final virus titre in blood meal was 10^8^ FFU/mL corresponding to the viral load encountered in some patients [17,18]. Feeders were maintained at 37°C and placed on top of the mesh of a plastic box containing 60 females. After 15 min of feeding (to minimize the effect of virus degradation in the infectious blood meal), mosquitoes were cold anesthetized and sorted on ice. Fully engorged females were transferred to cardboard containers and maintained with 10% sucrose in an environmental growth-cabinet set at 28°C ± 1°C, 80% humidity, and a 16 h: 8 h light regime.

### Vector competence and virus titration

CHIKV infection, systemic infection and transmission were determined at 3, 5, 7, 10, 12, 14 and 20 days post virus exposure (DPE) for both species. Presence of CHIKV in bodies indicates a midgut infection, while the presence of virus in heads and saliva indicates a systemic (disseminated) infection and virus transmission, respectively. Saliva collection was performed using the forced salivation technique [19]. At each time point, batches of mosquitoes from each species were anesthetized at 4°C before to have their wings and legs removed under a magnifying glass. Proboscis were then inserted into a 100 µL filter micropipette tip filled with 5 µL of heat-inactivated Fetal Bovine Serum (FBS). After 45 min, the medium containing the saliva was transferred into 1.5 mL tubes containing 45 µL of Leibovitz's L-15 medium (Gibco) and stored at −80°C. After salivation, mosquito heads were separated from the bodies and each compartment was grinded separately with 1 mm glass beads and 250 µL of Leibovitz's L-15 medium supplemented with 10% heat-inactivated FBS and 0.1% penicillin/streptomycin. Homogenization was realized in a grinder Precellys^®^24 for 2 × 30 s at 6000 rpm. All samples were frozen at −80°C before titration.

Virus titration was performed by visualizing infectious foci on a sub confluent culture of C6/36 cells by indirect immunofluorescence using 10-fold serial sample dilutions as previously described [20] with a minor modification: After fixation, cells were washed three times in Phosphate-Buffered Saline (PBS) and incubated for 1 hour at 37 °C with 50 μL/well of mouse ascetic fluid specific to CHIKV (primary antibody) diluted 1:1000 in PBS + 1% Bovine Serum Albumin (BSA) (Interchim, Montluçon, France). This mouse ascetic fluid was made by the Centre National de référence des arbovirus of the Institut Pasteur in March 2013 and provided by Pr. Despres Philippe.

### Statistical analysis

The time-dependent effect of the mosquito species on mosquito midgut infection, systemic infection and virus transmission was analysed by Firth's penalized likelihood logistic regression by considering each phenotype as a binary response variable. Penalized logistic regression, implemented through the *logistf* R package [21] was used to solve problem of separation that can occur in logistic regression when (i) the outcome has high prevalence, (ii) when all observations have the same event status for a combination of predictors or (iii) when a continuous covariate predict the outcome too perfectly. A full-factorial generalized linear model that included the time post virus exposure and the mosquito species was fitted to the data with a binomial error structure and a logit link function. Statistical significance of the effects was assessed by an analysis of deviance.

Virus titres in mosquito’s bodies, heads and saliva were compared between species by a Mann–Whitney-Wilcoxon rank sum test stratified on the time post-exposure as implemented in the *wilcox_test* function from the *coin* R package [22]. The effect of time post exposure on each quantitative phenotype for each species was assessed using a Kruskal–Wallis rank sum test. Time post exposure was converted into ordered factors to implement both tests.

The intra-host dynamic of systemic infection was assessed by a global likelihood function for each mosquito species as described in Fontaine et al*.* [23]. Probabilities of systemic infection at each time point post virus exposure were estimated with a 3-parameter logistic model. The probability of systemic infection at a given time point (*t*) is governed by *K*: the saturation level (the maximum proportion of mosquitoes with a systemic infection), *B*: the slope factor (the maximum value of the slope during the exponential phase of the cumulative function, scaled by *K*) and *M*: the lag time (the time at which the absolute increase in cumulative proportion is maximal). For easier biological interpretation, *B* was transformed into Δt, which correspond to the time required to rise from 10% to 90% of the saturation level with the formula: Δt = ln (81) / *B.* For each mosquito species, the *subplex* R function [24] was used to provide the best estimates of the three parameters to maximize the global likelihood function (i.e. the sum of binomial probabilities at each time point post virus exposure). This method accounts for differences in sample size when estimating parameters values.

### Haplotype network and phylogenetic analyses

Mitochondrial DNA (mtDNA) was extracted from single *Ae. geniculatus* specimens homogenates from Tirana (Albania) and Paris (France) using the NucleoSpin 96 Tissue Core Kit (Macherey-Nagel, Düren, Germany) according to the manufacturer’s instructions. The *Ae. geniculatus* specimen from Albania belonged to the population that has been exposed to CHIKV in this study. PCR amplification was carried out along a 566-bp region of the cytochrome oxidase I gene (COI) gene with primers C1-J-1718mod (5’- GGWGGRTTTGGWAAYTGAYTAG -3’) and C1-N-2191mod (5’- AGHWCCAAAAGTTTCYTTTTTCC -3’) (adapted from Simon et al. [25]), corresponding to region 1531- 2096 of the *Ae. aegypti* mtDNA (NC_035159) sequence [26]. Amplicons were cleaned using GenElute™ 96 Well PCR Clean-Up Kit (Sigma-Aldrich, St-Louis, MO, USA) before sequencing. Amplicons sequencing reactions were performed by using Big Dye Terminator v1.1 cycle sequencing kit (Applied Biosystems, Foster City, California, United States) and purified by ethanol precipitation. Cycle sequencing was performed on ABI3730XL sequence analyser (Applied Biosystems) using both the forward and reverse primers to create a consensus sequence and increase haplotype reliability. COI sequences from the two specimens were submitted to the identification tool of BOLD (Barcoding of Life) [27].

A set of 36 COI sequences from *Ae. geniculatus* and 2 COI sequences from *Ae. albopictus* were obtained from GenBank. An *Aedes echinus* COI sequence (GenBank accession number: MK070853) was kindly provided by Dr. Andreas Krüger from the Bernhard-Nocht-Institute for Tropical Medicine (BNITM), Hamburg, Germany. COI sequences from our two specimens (GenBank accession numbers: MK796909, MK796910) were aligned to the sequences retrieved from GenBank using ClustalW v.2.0.12 [28]. All COI sequences excepting both from *Ae. albopictus* were then imported in nexus format to the PopArt program [29] to create a TCS haplotype network using a 437 bp COI section covered by all 39 sequences from *Ae. geniculatus* and *Ae. echinus* mosquitoes.

Representative COI sequences (i.e. unique haplotypes over the full COI sequences) were analysed with RAxML v.8.2.10 [30] to generate the best-scoring maximum-likelihood (ML) tree of 10 runs with 100 thorough bootstrap replicates to infer the reliability of the branches. Topology was rooted to an *Ae. albopictus* (MG198601) COI sequence. The GTR nucleotide substitution model was chosen from a list of substitution models implemented in RAxML based on corrected Akaike’s Information Criterion (AICc) value using the PartitionFinder v2 software [31] with the linked branch length option. Phylogenetic trees were visualized using FigTree v.1.4.3 [32].

## Results

**Figure 1.**
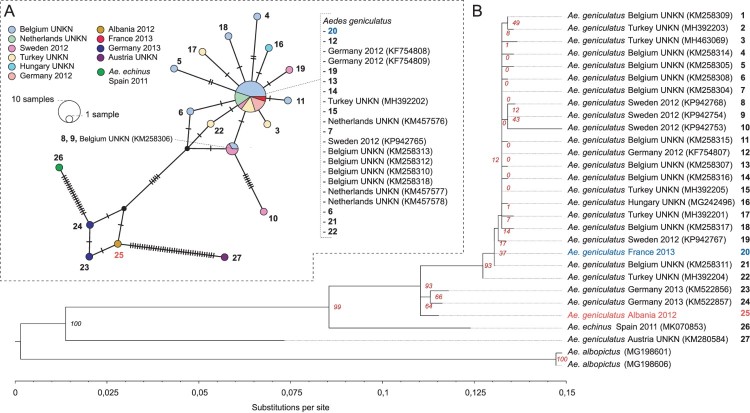
Cytochrome oxidase I gene (COI) sequence variation at the intraspecific level among European *Ae. geniculatus* mosquitoes. A – Haplotype network inferred by the TCS method using a 437 bp mitochondrial DNA sequence (COI gene) covered by 39 specimens identified as *Ae. geniculatus* or *Ae. echinus*. The size of the each circle represents the frequencies of the haplotype, with each colour showing the geographical origin and collection date of the specimen. The term UNKN was display when the collection date was not known. Mutations are shown as perpendicular bars along the branches and black small circles represent inferred unsampled haplotypes. Haplotypes in common with the maximum-likelihood phylogenetic tree (B) are represented with numbers. B – Phylogenetic relationships between unique COI haplotypes from *Ae. geniculatus*, *Ae. echinus* and *Ae. albopictus* mosquitoes. Inferences were calculated with a maximum-likelihood method implemented in RAxML v.8.2.10 [30]. Topology was rooted to the *Ae. albopictus* (MG198601) COI sequence. Bootstrap support values are indicated on each node in red. COI sequences from single *Ae. geniculatus* specimens from Tirana, Albania (mosquito population that was used in this study) and Paris, France are represented in red and blue, respectively. GenBank accession numbers are displayed for each sequence.

The COI sequence from one specimen from the Albanian population of *Ae. geniculatus* that has been exposed to CHIKV in this study has been sequenced. This COI sequence matched a Dahliana geniculata (an alternative name for *Ae. geniculatus* based on Reinert et al. [33]) sequence with 100% similarity and 100% probability of species assignment. This bold entry has an “early-release” status at the time of accession with no information excepting a rough location in Central Europe (most probably Germany). This sequence clusterized with two other COI sequences from Aedes specimens collected in Germany deposited in GenBank as *Ae. geniculatus* [34] (Figure 1). While being very closed from the *Ae. geniculatus* genetic cluster, this cluster can be distinguished from the Ae. echinus specimen on both the haplotype network (Figure 1(A)) and phylogenetic analyses with a high branch support (Figure 1(B)). Interestingly, a COI sequence from a specimen collected in Austria and identified as *Ae. geniculatus* (GenBank accession number: KM280584) clustered apart from all other sequences. The COI sequence of our *Ae. geniculatus* specimen from Albania, matched an *Ae. geniculatus* sequence with 100% similarity and 100% probability of species assignment. This specimen clustered with *Ae. geniculatus* mosquitoes collected all over Europe.

A total of 134 *Ae. geniculatus* and 124 *Ae. albopictus* engorged females were analysed, considering the mosquito mortality during the experiment. Indeed, 16 and 17 females died prior to sampling, respectively for *Ae. geniculatus* and *Ae. albopictus*. High midgut infection prevalences (>85%) were obtained for both species from the first time point after virus exposure. With 100% midgut infection among the exposed mosquitoes, *Ae. geniculatus* was highly susceptible to CHIKV infection (Table 1, Supplementary figure 1). Midgut infection prevalences were not influenced by the time post exposure (analysis of deviance, *p*-value = 0.0659) and a significantly higher midgut infection prevalence was observed in *Ae. geniculatus* vs *Ae. albopictus* (analysis of deviance, *p*-value = 0.0438).

**Table 1.** Body infection rate (BIR), systemic infection rate (SIR) and transmission rate (TR) at different days post-infection of *Ae. geniculatus* and *Ae. albopictus* exposed to CHIKV.

	*Ae. geniculatus*		* *	*Ae. albopictus*		
Day pi	BIR^a^	SIR^b^	TR^c^	BIR^a^	SIR^b^	TR^c^
3	100% (20)	15% (20)	0% (3)	100% (20)	90% (20)	27.8% (18)
5	100% (20)	20% (20)	0% (4)	95% (20)	100% (19)	63.1% (19)
7	100% (18)	72.2% (18)	30.8% (13)	100% (18)	100% (18)	38.9% (18)
10	100% (18)	72.2% (18)	38.5% (13)	100% (18)	100% (18)	27.8% (18)
12	100% (18)	72.2% (18)	84.6% (13)	100% (17)	100% (17)	23.5% (17)
14	100% (19)	78.9% (19)	73.3% (15)	93.3% (15)	100% (14)	28.6% (14)
20	100% (21)	100% (21)	61.9% (21)	87.5% (16)	100% (14)	14.3% (14)

Notes: Mosquitoes were contained in cardboard boxes after virus exposure. For all surviving mosquitoes per box and time point, we measured three parameters describing vector competence: (i) mosquito infection as measured by the presence of viral infectious particles in the body (abdomen and thorax): BIR, (ii) virus dissemination as measured by the presence of viral infectious particles in mosquito head: SIR, and (iii) transmission efficiency as measured by the number of mosquitoes with viral infectious particles in their saliva: TR. The number of mosquitoes analysed is displayed in brackets.

^a^Per cent of mosquitoes with infected body among all exposed mosquitoes.

^b^Per cent of mosquitoes with infected head among body infected mosquitoes.

^c^Per cent of mosquitoes with infectious saliva among mosquitoes with a systemic infection (mosquitoes that disseminate the virus beyond the midgut barrier).

CHIKV titres in bodies were significantly higher in *Ae. geniculatus* vs *Ae. albopictus* (stratified Mann–Whitney-Wilcoxon rank sum test, *p*-value < 2.2e-16), with approximately one log _10_ difference between values average across time points (5.1 vs 4.2 log 10 FFU/mL for *Ae. geniculatus* and *Ae. albopictus*, respectively). Virus titres in bodies were significantly different across time points post virus exposure in both species (Kruskal–Wallis rank sum test, *p*-value = 0.0002402 and 2.984e-15 for *Ae. geniculatus* and *Ae. albopictus*, respectively).

**Figure 2.**
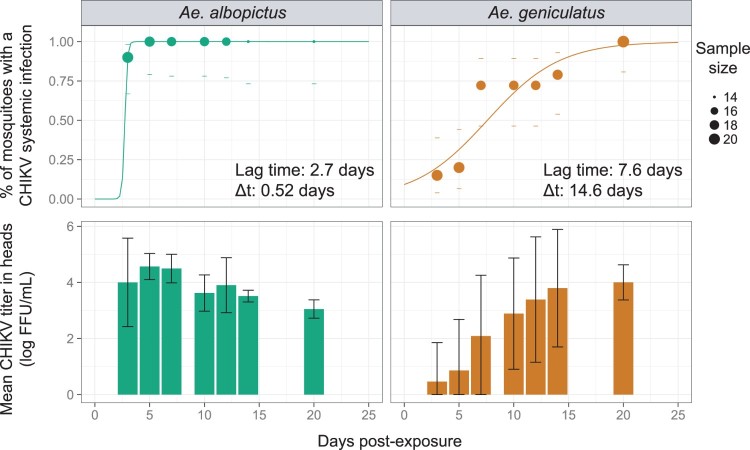
Systemic infection kinetic for *Ae. albopictus* and *Ae. geniculatus* species infected with CHIKV. The upper panel shows the cumulative prevalence of systemic infection over time post CHIKV exposure. Data points represent the observed prevalence at each time point with their size being proportional to the sample size. Dashes represent the 95% confidence interval of the prevalence. The fitted values obtained with a 3-parameter logistic model are represented for each species by a coloured line. The lag time and rising time estimates are represented for each species. The lower panel shows CHIKV titres in mosquito heads measured at each time points after oral exposure to the virus.

Systemic infection, as measured by the head infection prevalence, was significantly influenced by the time post exposure (analysis of deviance, *p*-value = 5.43e-13) and the mosquito species (analysis of deviance, *p*-value = 4.10e-15) but the time post exposure effect was not significantly different across the mosquito species according to logistic regression (analysis of deviance, interaction term, *p*-value = 0.6). The intra-host dynamic of systemic CHIKV infection (SIR values in Table 1) was quantified in both mosquito species by fitting a 3 parameters logistic model that assumes a sigmoidal distribution of the cumulative proportion of mosquitoes with a systemic infection over time. Both species presented a saturation level (K, or maximum proportion of infected mosquitoes with a systemic infection) equal to 100% (Figure 2). However, this saturation level was achieved later for *Ae. geniculatus* than for *Ae. albopictus*. Indeed, an estimated 14.6 days were needed for *Ae. geniculatus* to go from 10% to 90% of the saturation level whereas the saturation level was reached in less than one day for *Ae. albopictus*. The lag time M, which can represent a proxy for the Extrinsic Incubation Period-50 (EIP-50) – the time required for transmission prevalence to reach 50% of the saturation level – was 2.7 days in *Ae. albopictus* mosquitoes compared to 7.6 days in *Ae. geniculatus* (Figure 2).

CHIKV titres in heads were significantly lower in *Ae. geniculatus* vs *Ae. albopictus* (stratified Mann–Whitney-Wilcoxon rank sum test, *p*-value = 3.3e-4), with approximately 1.3 log_10_ difference between values average across time points (2.5 vs 3.8 log 10 FFU/mL for *Ae. geniculatus* and *Ae. albopictus*, respectively). Virus titres in heads were also significantly different across time points post virus exposure in both species (Kruskal–Wallis rank sum test, *p*-value = 2.621e-07 and 1.781e-10 for *Ae. geniculatus* and *Ae. albopictus*, respectively).

Virus transmission prevalences were measured by assessing the presence of the infectious virus in mosquito saliva. Virus transmission prevalences were significantly different between mosquito species (analysis of deviance, *p*-value = 0.0038) and the effect of time post-exposure varied significantly between mosquito species (analysis of deviance, interaction term, *p*-value = 3.05e-4). It was not possible to model the cumulative virus transmission prevalence over time for the *Ae. albopictus* species because of a lack of saturation level. Proportion of virus in saliva clearly collapsed from day 5 post virus exposure (Table 1, Supplementary figure 2). The same phenomenon could be observed for *Ae. geniculatus*. However, the 3-parameter model could nonetheless fit the data, giving the following estimates: 70% of saturation level, an EIP-50 of 8 days and a rising time of 6.2 days (Supplementary figure 2).

CHIKV titres in saliva were significantly higher in *Ae. geniculatus* vs *Ae. albopictus* (stratified Mann–Whitney-Wilcoxon rank sum test, *p*-value = 1.8e-3), with 0.24 log _10_ difference between values average across time points (0.51 vs 0.27 log 10 FFU/mL for *Ae. geniculatus* and *Ae. albopictus*, respectively). Titres in heads were significantly different across time points after virus exposure in mosquitoes from the *Ae. geniculatus* species only (Kruskal–Wallis rank sum test, *p*-value = 4e-03 and 0.14 for *Ae. geniculatus* and *Ae. albopictus*, respectively). Virus titres in saliva were clearly increasing over time in mosquitoes from the *Ae. geniculatus* species.

## Discussion

Chikungunya virus (CHIK) is one of four arboviruses with dengue virus (DENV), Zika virus (ZIKV) and yellow fever virus (YFV) that can be sustained in a human-vector-human transmission cycle. All four originated in wild primates but can cycle in the urban environment, transmitted by peridomestic mosquitoes. Intercontinental travel and trade were historically involved in the transmission of these viruses world-wide, sometimes involving recruitment of new local vectors [35,36]. For instance, the YFV, originating in Africa, was introduced to the Americas during the slave trade, where it entered new transmission cycles involving autochthonous arboreal species, such as Haemagogus janthinomys and mosquito from the Sabethes genus, and non-human primates in primary tropical rain forest. The virus then regularly propagated from the jungle into urban transmission cycles [37].

Europe is the world’s leading tourism destination with 671 million arrivals (50% of all international tourists) in 2017, a number that continues to increase [38]. Travellers with arbovirus infections [39–45], including CHIKV [46] have initiated autochthonous transmission [5,6] vectored by the invasive mosquito *Ae. albopictus* species. At the same time, Europe harbours native mosquito species potentially able to transmit emerging arboviruses. Yet, only two studies [47,48] have assessed the potential for CHIKV infection in five indigenous species from the south of France and Italy (*Culex pipiens*, *Aedes caspius*, *Aedes detritus*, *Aedes vexans*, and *Anopheles maculipennis*)[49]. The three *Aedes* species were susceptible to virus infection but the transmission potential of infectious viral particles in the mosquito saliva was not investigated. These insects represent a small portion of all European anthropophilic mosquitoes.

Our study has revealed the ability of the European mosquito *Ae. geniculatus* to transmit CHIKV experimentally. Vector competence of *Ae*. *geniculatus* for CHIKV was compared to the vector competence of an *Ae. albopictus* population originating from Albania, the first European nation that has been reported to be colonized by this species. Aedes geniculatus was revealed to be highly susceptible to CHIKV infection. Midgut infection by an arbovirus is achieved very rapidly after exposure to the infectious blood meal when infectious virus particles are still in contact with the midgut wall cells. Virus dissemination in secondary tissues can only occur in mosquitoes with infected midgut and is, in contrast to the infection phenotype, a dynamic process scaled in a day unit that can have epidemiological significance. Variation in DENV dissemination dynamics in mosquitoes was reported to significantly affects the risk and magnitude of dengue outbreaks [23]. We revealed that both species were completely able to disseminate CHIKV in their secondary tissues. However, this 100% saturation level of infected mosquitoes with a disseminated infection was reached in *Ae. albopictus* 15 days earlier than *Ae. geniculatus.* The estimated virus dissemination lag time, that can be considered as a proxy for the EIP-50 (extrinsic incubation period in days until 50% of maximum infectiousness), was nearly 8 days for *Ae. geniculatus* whereas is was less than 3 days for *Ae. albopictus.* Combined with vector longevity, EIP is the most powerful contributor to vectorial capacity according to the Ross-MacDonald equation [50]. Vectorial capacity is a restatement of the basic reproductive rate (R0) of a pathogen and define the number of secondary infections expected to occur from the introduction of a single infection in a naive population [51]. The longer it would take for a virus to disseminate from the infected midgut to the saliva, the fewer opportunities the mosquito would have to transmit the virus to a human host before its death. For an equal vector longevity and gonotrophic cycle duration, *Ae. albopictus* can thus be considered as a better vector than *Ae. geniculatus*. In other words, *Ae. albopictus* mosquitoes would infect more susceptible human hosts before they died than *Ae. geniculatus* because they would achieve an infectious status sooner. As a result of these intra-host systemic infection dynamic difference among mosquito species, the probability of outbreak initiation and the number of human cases would be lower in the field [23] when *Ae. geniculatus* only is present, as compared to *Ae. albopictus*. This theory can be thwarted if *Ae. geniculatus* mosquito’s life span is longer than *Ae. albopictus* one [52]. *Aedes geniculatus* might then be involved in autochthonous CHIKV transmissions in Europe in complementation with or in absence of *Ae. albopictus*. The higher CHIKV titres observed in *Ae. geniculatus* saliva as compared to *Ae. albopictus* can mirror a higher infectiousness for the European native species. The impact of virus titre in mosquito vector saliva in initiating an infection in the human host and in influencing the intrinsic incubation period is not known. This has never been studied because of the difficulty to carry out dose-response experiments in human hosts or animal models. In addition, possibility of rapid virus adaptation to new vectors was already observed [53,54]. CHIKV evolution toward restricted EIP duration in this autochthonous species would greatly increase the epidemic potential of this vector-virus couple.

The Albanian population of *Ae. albopictus* was highly susceptible to CHIKV with 100% of systemic infections achieved before the fifth days post virus exposure. Fast CHIKV disseminations were already reported at equivalent infectious blood titres for several *Ae. albopictus* populations. CHIKV infectious particles were shown to be present in salivary glands or other peripheric organs as soon as 2 days post virus exposure [19,55]. These really fast extrinsic incubation periods and high systemic infection saturation levels for several European *Ae. albopictus* populations [47,56–58] further confirm the risk for CHIKV emergence in Europe [6,59,60]. Vector competence dynamic for a virus/mosquito species combination is strongly influenced by the infectious blood meal titre (virus dose), the environmental conditions, the time of mosquito sampling post virus exposure, sample sizes from which proportions were inferred, or the genetic background of the viruses, their mosquito vectors and their interaction [61]. This lack of standardization across vector competence studies impedes direct comparison of results. Modelling vector competence dynamic for a set of parameters can provide a holistic view of the impact of time on vector competence and give reference estimates that can help direct result comparison across studies.

The *Ae. geniculatus* species is known under different names according to classifications. We use the species denomination from Knight and Stone’s catalog [62]. This species was later reclassified at the genus level by Reinert and colleagues [33], but we considered the revised species name *Aedes (Dahliana) geniculatus* from the stable classification of the tribe Aedini [63]. The following species denomination can be reported in the literature to describe the same specimens: *A. geniculatus*, *Ochlerotatus geniculatus* or *Dahliana geniculata* [64,65]. *Aedes geniculatus* specimens that were assessed for their vector competence for CHIKV in this study were genetically closed to 2 specimens collected in Germany in 2013 [34] (Figure 1). This cluster of sequence was genetically separated from other described *Ae. geniculatus* species and from the *Ae. echinus* specimen, as represented by a Spanish sequence collected in 2011 [34]. Another specimen collected in Austria (KM280584) and submitted in GenBank as *Ae. geniculatus* (Figure 1) appears to be genetically divergent from all other specimens. Three mosquito species from the subgenus “Finlaya” (Theobald, 1903) can be considered as the closest relative species of *Ae. geniculatus*, namely *Ae. echinus*, and *Aedes gilcolladoi* [34]. Our morphological determination of the *Ae. geniculatus* species was done as follow. At the adult stage, the female of *Ae. geniculatus* is morphologically indistinguishable from *Ae. gilcolladoi* specimens. While being morphologically very close to *Ae. echinus* mosquitoes, *Ae. geniculatus* adults females specimens can be distinguished from the former species by the absence of a pale scaly spot on the metameron [12]. In addition, it is possible to distinguish *Ae. geniculatus* from both other species at the larval stage by the ramification of most of the silks present on the abdominal segments IV to VII. Indeed, *Ae. echinus* and *Ae. gilcolladoi* have rigid silks generally with 7 or more branches while *Ae. geniculatus* specimens have rigid silks with less than 6 branches [12]. These determination criteria were verified for adults and larvae from the mosquito population used in our study. As discussed by Krüger and colleagues, it is possible that the specimen identified as *Ae. echninus* in Spain (COI GenBank accession number MK070853) belongs to the *Ae. gilcolladoi* species. We can also make the assumption that the Austrian specimen identified as *Ae. geniculatus* (COI GenBank accession number KM280584) belongs to the *Ae. echinus* species and that our Albanian specimens are representatives of a cryptic sibling species of *Ae. geniculatus*. Because no or few public reference DNA sequences are available for these species, it has yet to be determined if these different genetic clusters belong to distinct species or represent a different sub-species inside a complex. The vector competence dynamic values that are reported in this work might not be representative of other relative species, or sub-species from a hypothetical *Ae. geniculatus* complex.

The biology of *Ae. geniculatus* is poorly studied [10]. From what is known, the species can bite aggressively both humans and animals mainly during daylight hours [10] and personal observation. Its larvae are commonly found in mature trees’ holes [66]. Adults often coexist with *Ae. albopictus* in Europe but population densities of *Ae. geniculatus* generally didn’t reached those of *Ae. albopictus* in peri-urban areas. More data are needed to fully characterize the level of contact with human hosts. As an anecdote, we captured *Ae. geniculatus* on Jim Morrison grave, one of the most visited graves by tourists of all nationalities in Pere Lachaise cemetery, in the centre of Paris, where millions of people come every year, strengthening the threat for this species in local virus transmission. Its flight dispersion is not considered superior than *Ae. albopictus*. Consequently, this species would not carry viruses outside of the area treated in the frame of the vector control intervention. Arbovirus vertical transmission is a rare event, and its occurrence hazard is correlated with vector density and the surveillance of virus. If higher CHIKV vertical transmission rates are achieved in *Ae. geniculatus* species, vertical transmission of CHIKV in overwintering *Aedes* mosquitoes might contribute to the maintenance of this virus during winter. Populations of *Ae. geniculatus* are generally monovoltine (i.e. one generation per year), and their diapause termination is asynchronous [10], enabling a reintroduction of the virus later in the following year when the population of the primary vector *Ae. albopictus* is already high. It seems therefore necessary to improve the knowledge on this species biology, including longevity, flight and host-seeking behaviour, diapause and virus overwintering to fully assess its epidemic potential. At last, temperature is strongly influencing vector competence. It can be of interest to compare vector competence for CHIKV between both mosquito species at lower temperatures.

Our study has several limitations. First, our estimates of virus dissemination dynamic can be biased. They rely on the modelization of the measured cumulative proportion of mosquitoes with a disseminated infection over time. Concerning *Ae. geniculatus*, the modelized proportion is not null at the time of mosquito exposure to the virus as it should be. The number and repartition of time points and the proportions accuracy (that depend on sample size at each time point) are influential in determining accurate parameter estimates of the virus dissemination dynamic. This method can nonetheless provide realistic estimates and offer the possibility to consider the dynamic nature of vector competence. Importantly, this allows to disentangle the incubation period effect from the maximum proportion of systemic virus infection (i.e. the maximum proportion of mosquito with a systemic infection is similar for both species but is reached later for *Ae. geniculatus*. An assessment of systemic infection prevalence at an early single time point would have conclude to differences in dissemination rates between species). Then, the transmission dynamic estimates must be considered with caution because the method to detect viruses in mosquito saliva do not distinguish true negatives from negatives resulting from mosquito that did not expectorate saliva. This might explain the observed decrease of transmission rates for *Ae. albopictus*. Because lower virus titres were observed in *Ae. albopictus* saliva as compared to *Ae. geniculatus*, false negative virus detection might have arisen more frequently in *Ae. albopictus* saliva as compared to *Ae. geniculatus* saliva samples due to a detection threshold issue. Alternatively, this decrease could be explained by the fact that infected mosquitoes died before non-infected mosquitoes or that oldest mosquitoes begin to clear the infection through immune function [67] or by the natural death of virions. A difference in the decline of viral titre of CHIKV over time was already shown between *Ae. albopictus* and *Ae. aegypti*, suggesting a species-specific interaction with the virus [68]. It can, however, be concluded that both species can transmit infectious CHIKV particles as soon as 3 and 7 days post exposure for *Ae. albopictus* and *Ae. geniculatus*, respectively.

CHIKV is in expansion throughout the world and Europe is not spared. Since the CHIKV outbreak in Northern Italy in 2007, and more recently the isolated cases of chikungunya and dengue recorded in France and Croatia from 2010 to 2013 [40,69], it has been acknowledged that Europe is vulnerable to local transmission of “tropical” arboviruses. Epidemics risk is in connection with the steady increase of imported cases of *Aedes*-bornes viruses as well as with the European expansion of *Ae. albopictus* [70]. So far, studies on the epidemiology of the arboviral diseases chikungunya and dengue in European countries have focused on the invasive “Asian tiger mosquito” without considering the potential role for indigenous vector species. *Aedes geniculatus* and other potential vector species are, to this day, absent from epidemiological models so far [47,58]. These results show the importance of considering European indigenous species to assess overall risk of arbovirus transmission in Europe. We suggest that *Ae*. *geniculatus* species should be considered as a potential secondary vector in the Palearctic region.

Assessing the vector competence of the different European mosquito species, but also other regions around the world potentially exposed to arboviral outbreak, for other arboviruses or other strains, will help to anticipate patterns of transmission of arbovirus as CHIKV, ZIKV and DENV and the relative contribution of different vector species to virus’s amplification and persistence (transmission and reservoir) in these areas. Moreover, we recommend to monitor on the field all mosquito species, whether native or invasive, during surveillance programmes on future outbreaks to further characterize the autochthonous mosquito fauna that can be potentially involved in arbovirus transmission.

## Supplementary Material

Supplemental Material
